# Similar image retrieval in large-scale trademark databases based on regional and boundary fusion feature

**DOI:** 10.1371/journal.pone.0205002

**Published:** 2018-11-15

**Authors:** Meihong Wu, Wenbin Xiao, Zhiling Hong

**Affiliations:** 1 School of Information Science and Technology, Xiamen University, Fujian, China; 2 Institute of Digital Fujian Big-Data in Higher Education, Xiamen University, Fujian, China; Case Western Reserve University Jack Joseph and Morton Mandel School of Applied Social Sciences, UNITED STATES

## Abstract

In order to retrieve similar trademarks from large-scale trademark databases, combining the characteristics of trademark images, this paper presents a trademark image retrieval method based on regional and border feature fusion. Based on the target image extraction, the proposed approach describes the target region and border features. The region feature description is mainly based on the concept of partition block statistics. The region is divided into equal-area unit using concentric circles, and feature extraction is performed in each small block unit. For the border feature description, this study first detect corners, and then construct a Delaunay graph and extract features by combining the corner detected and the Delaunay triangulation reconstruction. In the search process, the method also incorporates information such as the trademark's color characteristics, trademark classification, and trademark keywords. The present study carried out image retrieval experiment on CE-SHAPE-1 database containing 1400 MPEG-7 core experimental shape, a classification trademark database containing 2000 images, and a national trademark database containing approximately 4.89 million images. The experimental results show that the proposed approach combines the advantages of region and border feature description, and can choose the best among various local optimizations, which makes the retrieval result more effective, more in line with human visual perception, and improves the retrieval accuracy.

## Introduction

With the quick development of trans-nation and trans-regional economy in recent years, the number of trademarks has been increasing year by year. Take China as an example. According to the bureau of industry and commerce, the total number of applied trademarks was 3.81 million at 2016, and it grows to 5.91 million by the end of 2017, which increased by 29.19% year-on-years. At the same time, China’s total trademark applications and registration applications have ranked first in the world for sixty consecutive years[1]. The number of registered trademarks is very large, and because of this judging whether or not a to-be registered trademark is similar to a registered one is not only laborious but also very inefficient.

There are many kinds of trademarks, whose images vary widely. Generally trademarks can be divided into the following three types[1, 2]: Text mark, which refers to the mark containing only words; Figurative mark, which refers to the mark containing images or geometric figures; Associated mark, also known as a composite trademark, refers to the marks containing two or more components. Relatively speaking, the management of word marks is easier and we can search this kind of marks by text annotations. Thus the main problem with trademark management is the existence of trademarks containing images, which are the vast majority of the registered trademarks. This article focuses on the research of trademarks containing images.

Trademark image retrieval is one of the important means to implement trademark search, which examine the repeatability and similarity of trademark images. According to the trademark search mechanism, the trademark image retrieval technology can be divided into three types: classification search, text retrieval and content-based retrieval. The classification search method indexes the image using the Vienna classification code[1] proposed by the World Intellectual Property Organization. This method divides the logo image elements into 29 categories, about 111 subcategories and 1569 subdivisions. This method has disadvantages such as time-consuming, artificial classification subjectivity, fixed classification structure, etc. The text retrieval method utilizes the advantages of natural language to accurately describe the image content in words, reveals its internal semantic relationships, forms a descriptive free text, and then establishes the index to achieve the matching of the search keywords and the feature mark. The essence of this method is the string matching principle. Text retrieval methods also have drawbacks such as large manual workload, inadequate description, and description subjectivity[3]. The content-based retrieval technology obtains its content and retrieves content based on the process of processing, analyzing and understanding the image from the bottom layer to the high layer. The method calculates the visual features of each image in the database, such as color, texture, and shape, to form the feature vectors. In the search, we first calculate the feature vector of the query image by the same method, and then we calculate the distance between the image feature vector and each image feature vector in the database, and at last we retrieve an image similar to the query image according to the distance. It can effectively overcome the problems of the above two methods.

The principle of trademark image design is concise, and having a clear theme. Trademark image design has the characteristics of simple shape, monotonous color, size comparison rules, etc, and based on this, the retrieval of the trademark image mainly uses the shape information and color information[4]. Many scholars at home and abroad have conducted in-depth research about the trademark image retrieval method. The color information description related methods includes: statistical histogram, cumulative histogram and color layout, color-related graph, and color moment, etc.[5]. As an artificial image, the shape features of trademark image are more prominent than the color features, and many trademark images are monochrome non-textured binary images. Therefore, we generally use shape features for retrieval.

## Related work

There are two kinds of feature extraction methods for two-dimensional shapes in MPEG-7: border-based feature extraction methods and region-based feature extraction methods.

In terms of border-based feature extraction, Cortelazzo et al. [6] used chain code strings to describe the shape features of the trademark and string matching methods to measure the degree of similarity of the chain code strings earlier, but they did not consider the invariance of the transformation. Peng et al. [7] proposed similarity retrieval of trademark images using closed border information. The approach tries to decompose the trademark image into a set of multiple closed borders, but it does not consider the directional relationship of adjacent corner codes; On the basis of Peng, Yin et al. [8] analyzed the directional relationship of adjacent corner codes, proposed a string representation method of two-level boundaries, and performed trademark image retrieval by combining it with features such as region area and symmetry. Also, many scholars used color histograms and edge direction histograms as features to achieve the invariance of translation, scaling, and rotation changes [9–12]. But the color is just a specific attribute of the shape of the logo. Dissimilar shapes may have the same color, while similar shapes may have different colors. Therefore judging the similarity of trademarks should mainly depend on the shape characteristics.

In terms of region-based feature extraction methods, the moment-based method is the most commonly used one, which describes the statistical distribution characteristics of the regional pixels. Teh[13] compared various orthogonal moments and non-orthogonal moments and found that the performance of Zernike moment is superior. Goyal[14] used Zernike moments for trademark image retrieval, and the results are consistent with human visual perception. Lin[15] uses the distance-angle histogram as the feature vector for trademark retrieval and finds that it has better retrieval performance. In addition, such as used a two-dimensional Hidden Markov model method to perform the deformable trademark retrieval[16]; used wavelet transform to extract the edges of the image, and then used the improved moment feature to describe the edge of the image[17]; extracted the shape feature on the sub-block decomposed by the quad-tree algorithm, and made the method scale and rotation invariant through pre-processing steps[18], etc.

As can be seen from the literature, most of the past related work focused on a single type of feature, either based on borders or regions. The border-based method is not suitable for describing complex shapes and is sensitive to local variations, but it has a lower computational cost; region-based method requires a lot of calculations, but it can be used to describe complex shapes and is insensitive to local variations. Established a comprehensive feature shape description method by combining the two representation methods, we can perform trademark image retrieval more accurately and efficiently.

This paper combines the features of trademark image and presents a full-image trademark image retrieval method based on border and regional feature fusion (BR-MATCH). Based on the target image extraction, the proposed approach describes the target region and border features. The region feature description is mainly based on the concept of partition block statistics. The region is divided into equal-area unit using concentric circles, and feature extraction is performed in each small block unit. For the border feature description, we first detect corners, and then construct a Delaunay graph and extract features by combining the corner detected and the Delaunay triangulation reconstruction method. In the search process, the method also incorporates information such as the trademark's color characteristics, trademark classification, and trademark keywords.

We carried out image retrieval experiment on CE-SHAPE-1 database containing 1400 MPEG-7 core experimental shape, a classification trademark database containing 2000 images, and a national trademark database containing approximately 4.89 million images. The experimental results show that the proposed approach combines the advantages of region and border feature description, and can choose the best among various local optimizations, which makes the retrieval result more effective, more in line with human visual perception, and improves the retrieval accuracy. The remainder of this paper is organized as follows. We present BR-MATCH method in Section 2 and perform experimental verification and analysis in section 3. Finally we make a conclusion in Section 4.

## BR-MATCH method

**Fig 1:**
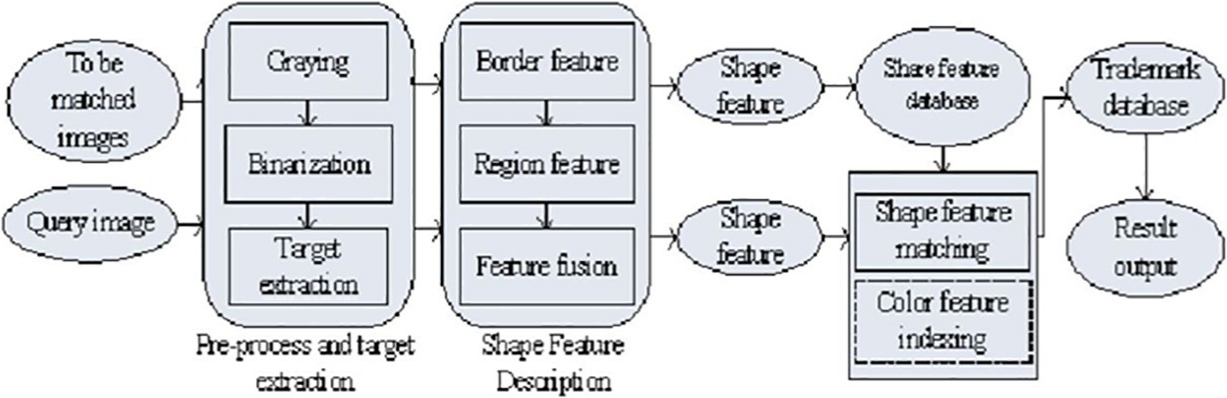
Trademark archive and search by BR-MATCH.

The process of archiving and retrieving trademark images using the BR-MATCH method is shown in Fig 1. As can be seen from the figure, the search process includes two pipelines, the image archiving process and the trademark search process. When archiving the images, we first analyze every of the matched images and then we extract shape feature, after this we add the shape feature vectors into the shape feature database, and the image into the image database at the same time. When retrieving an image, a similar process is performed. After the input query image is preprocessed and the target is extracted, we describe the target shape by features from two aspects respectively: the boundary and the regional. Then we combine the two aspects to form the overall shape feature description. Finally we match the feature description of the input query image and the features of the extracted shape feature database and output the corresponding trademark from the trademark database according to the matching score. The following describes the processing function modules in detail.

### 3.1. Preprocessing and image target region extraction

When we human-being observes an image, it is natural for us to divide the image into foreground and background. Human visual experience is different. For the same image, some people may be more sensitive to the global features of the image, while others are more interested in the local features of the image. In general, people's evaluation of computer search results is mainly measured by the objects (local features) in the image. For image retrieval, the object area is more in line with the visual experience of the human eye than the background area. Therefore, extracting image features from the object area is more suitable for content-based image retrieval. In order to describe the shape of the object area, the following three steps are needed: graying of the RGB image, binarization of the gray image, and image target region extraction. We will describe them separately below.

The trademark database often contains both color images and grayscale images, therefore we first make the RGB image to a gray one to make the extracted features have better applicability and unify the feature extraction process. Graying a color image is the process of equalizing the R, G, and B component values of the image. We can complete the graying process just by calculating Y = 0.299×*R*+0.587×*G*+0.114×*B*, and making R, G, B values equal to Y.

The binarization processing of the grayscale image is to select a threshold value and if the grayscale value of a certain pixel in the image is smaller than the threshold value, then we set it to be 0; otherwise, the grayscale value is set to 255. The key to the binarization lies in the selection of thresholds, which we use to distinguish the foreground and the background in the image. There are three kinds of threshold selection methods: global threshold, local threshold, and dynamic threshold. Since the trademark image belongs to an artificial image, the foreground and background are clearly separated, and its histogram distribution has a bimodal image. Therefore, we use a global threshold method to perform binarization. This paper uses the Otsu algorithm[19] for threshold selection.

After the image binarization processing, the image is divided into two parts: foreground and background, but further judgment should be made to determine which part is the foreground. First define the importance of every specific area G(*R*):

$$G\left(R\right)=1-\frac{\sum_{pixel\left(i,j\right)\in R}\max\left\{\frac{\left|2\times i-width\right|}{width},\frac{|2\times j-height}{height}\right\}}{N\left(R\right)}$$ (1)

Among them, *R* represents the specific area, *N*(*R*) is the number of pixels in the area. *G*(*R*) measures the distance between the area *R* and the image boundary. If the pixels in the area are mostly near the image boundary, then *G*(*R*)≈0; Otherwise we set *G*(*R*)≈1. In general, the center part of the image will receive more attention, so the above formula can reflect the degree of importance of the area to some extent.

Assume that the transformed binary trademark image is *f*(x, y), and its two types of regions are denoted by *f*_1_(x, y) and *f*_2_(x, y) respectively, we can calculate the importance of the two types of region *G*(*R*) by substituting them into formula (1). Since the object of the trademark is generally located at the center of the image, its *G*(*R*) value tends to be 1. We select the area corresponding to the larger value as the target pixel area.

### 3.2. Shape feature description

On the basis of obtaining the standardized target pixel area, the following three steps are needed for the shape feature description: border feature description, regional feature description, and feature fusion. We will describe them separately below.

#### 3.2.1 Regional features description

**Fig 2:**
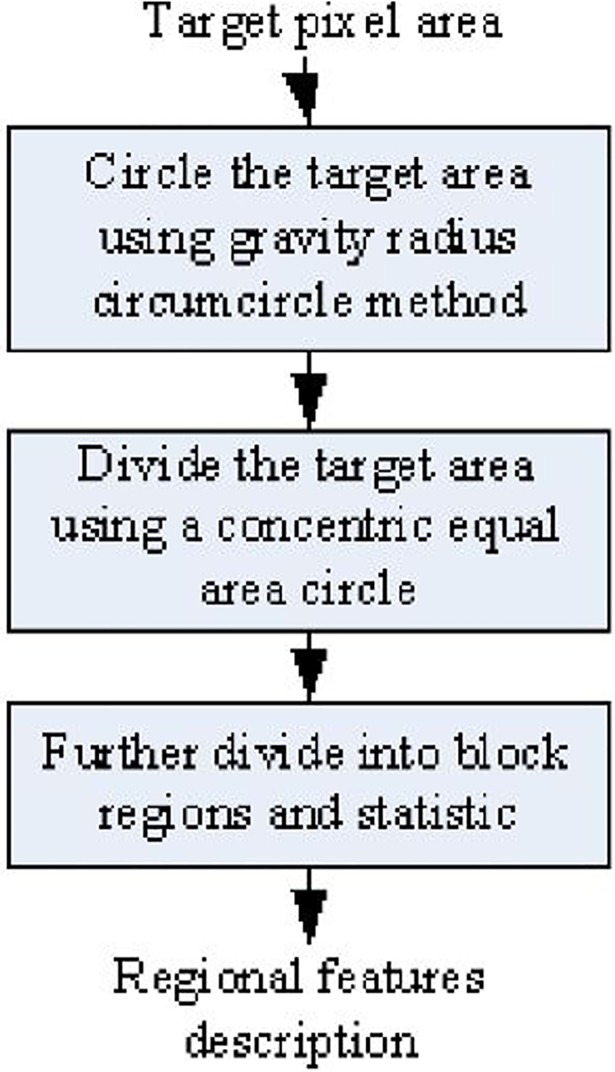
Region feature extraction.

The extraction process of regional features is shown in Fig 2. On the basis of obtaining the target pixel area, we first use the center of gravity radius circumcircle method to quickly circle the target area. Second we divide the target area using a concentric equal area circle division strategy by combining the original binary trademark image, and then we further divided each ring sub-regions into block regions. Finally we extract features of block region. The detailed description of the extraction process is as follows.

(1) Firstly, use the center of gravity radius circumcircle method to obtain the target area. Select the point of gravity $\left(\bar{x},\bar{y}\right)$ of the binary target area f(x, y) as a reference point:

$$\bar{x}=\frac{m_{10}}{m_{00}},\bar{y}=\frac{m_{01}}{m_{00}}$$ (2)

$$m_{pq}=\int_{-\infty }^{\infty }\int_{-\infty }^{\infty }x^{p}y^{q}f(x,y)dxdy$$ (3)

*m*_*pq*_ is the *p*+*q*-th moment of *f*(x, y). The point of gravity is selected because it is a global descriptor whose coordinates are calculated based on all the points belonging to the area. It is global.

Using the center of gravity as a reference point, we calculate the distance between each target pixel point in the image to the reference point. Clear the distance is not sensitive to the translation change of the target area. The distance from the pixel point to the center of gravity is:

$$d_{i}=\sqrt{{\left(x_{i}-\bar{x}\right)}^{2}+{\left(y_{i}-\bar{y}\right)}^{2}}$$ (4)

Set *D*_avg_ the average distance from all the target pixel points in the image to the reference point, then there are:

$$D_{avg}=avg(d_{i}),\;i=1,2,\cdots ,n$$ (5)

**Fig 3:**
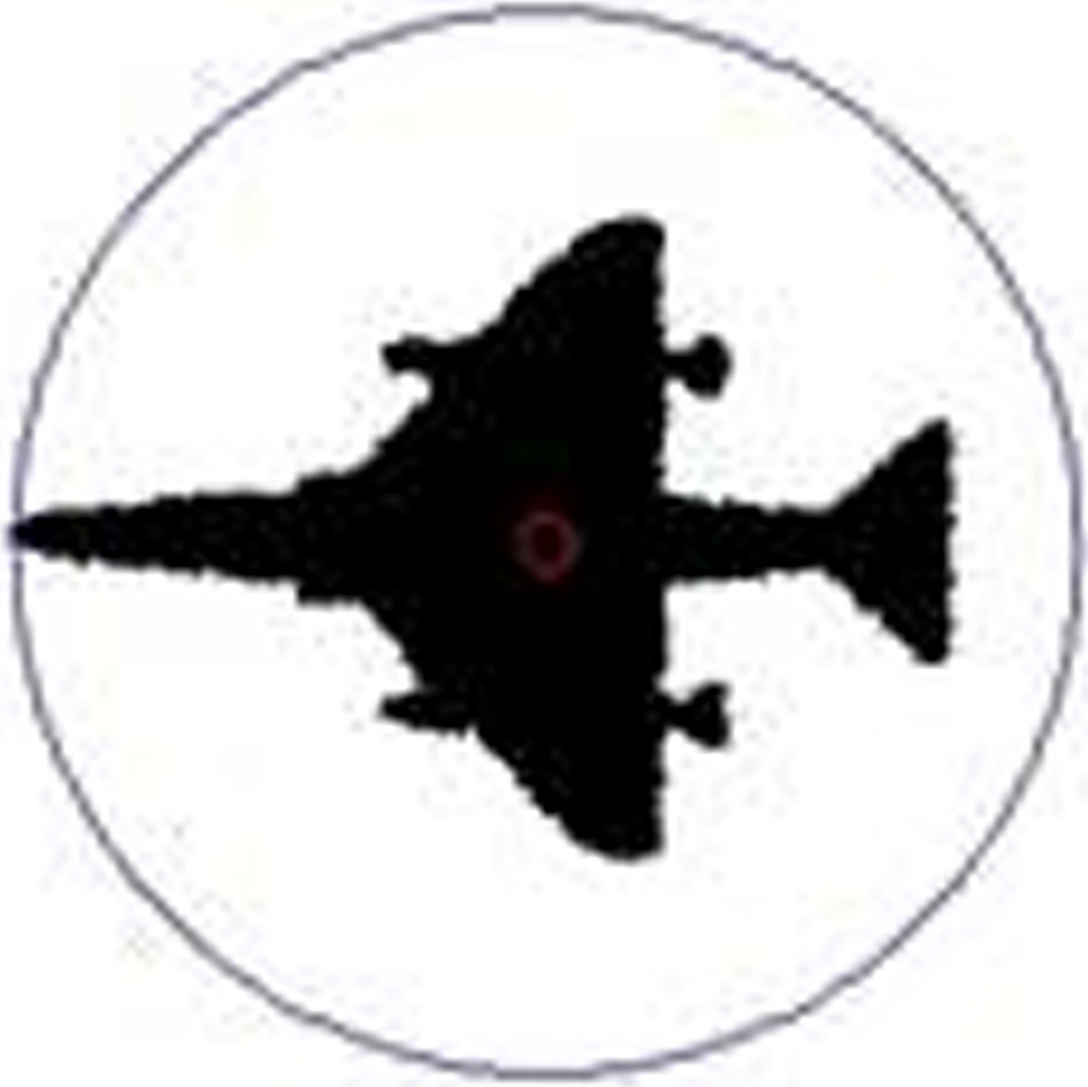
Circle the target area.

Make a circumscribed circle of the target pixel area with the center of gravity as the center of the circle, and 2 *D*_avg_ as the radius. Then we take the circle as the target area of the image. Fig 3 shows an example of extracting a circumscribed circle from a shape image and take it as the target area using the center of gravity radius circumcircle method.

(2) The second step is dividing the target area. Use concentric circles to divide the target area into *M* sub-zones:

$$c_{1}(x,y),\cdots ,c_{i}(x,y),\cdots ,c_{M}(x,y),1\leq i\leq M,c_{i}(x,y)\subset C(x,y)$$ (6)

where *c*_1_(x, y) represents for the center circle, *c*_2_(x, y)~*c*_M_(x, y) represent for multiple concentric rings. There are two methods for dividing the concentric circles in the circular target area: one is the equal-distance interval method, that is, the length of the distance covered by each sub-area is equal; the other is the equal-area method, that is, the area covered by each sub-area is equal. Based on our experiments, the difference in retrieval performance of these two partitioning methods is small, with the equal-area method slightly better. Therefore, in this article, we use the equal-area method to divide circular target area:

$$c_{i}(x,y)=\{\begin{array}{ll} \left\{(x,y)|{\left(x-\bar{x}\right)}^{2}+{\left(y-\bar{y}\right)}^{2}\leq \frac{r^{2}}{M}\right\} & i=1\\ \left\{(x,y)|\frac{(i-1)\times r^{2}}{M}<{\left(x-\bar{x}\right)}^{2}+{\left(y-\bar{y}\right)}^{2}\leq \frac{i\times r^{2}}{M}\right\} & 1<i\leq M \end{array}$$ (7)

**Fig 4:**
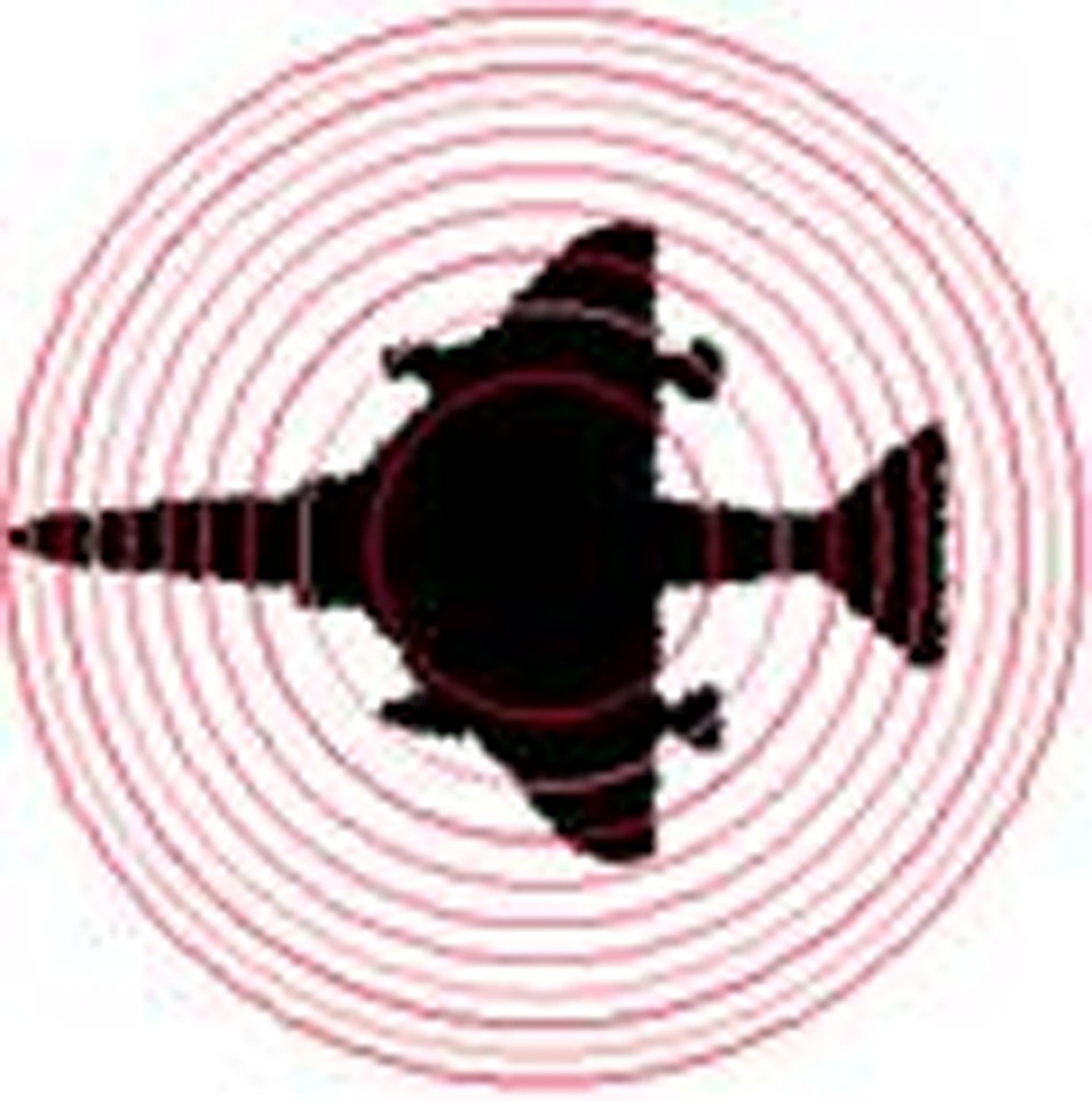
Concentric circle.

$\left(\bar{x},\bar{y}\right)$ is the center of the circumscribed circle, *r* is the radius, (*x*,*y*)∈*C*(*x*,*y*) and *C*(x, y) represent the area covered by the circumscribed circle. Fig 4 shows an example of equal-area method, where the area of each ring and the center circle is equal.

(3) Finally, do statistical analysis of the block area of the concentric ring. The block area statistical feature extraction method requires further subdivision of the concentric circles we obtain from the above step. For each ring sub-block, it is divided into equal parts radially, and then we conduct shape feature extraction for each sub block. The target pixel weight *t*_*i*_ of the sub-block reflects the number of target pixels in the *i*-th block, that is, *t*_*i*_ represent for the ratio of the number of target pixel points to the total number of pixel points in the *i*-th sub-block. Set *B*_*i*_ the block range of the *i*-th sub-block:

$$t_{i}=\frac{\sum_{(x,y)\in B_{i}}f(x,y)}{\sum_{(x,y)\in B_{i}}1}$$ (8)

If the weight of the target pixel in the *i*-th sub-block is less than a certain threshold *t*, then the pixels in the sub-block are likely to be noise points. To avoid the influence of the noise point, we set the statistical value of this area *b*_*i*_ to be 0; otherwise, we set *b*_*i*_ to be 1 for the efficiency requirements of shape retrieval comparisons. The specific calculation is as follows:

$$b_{i}=\{\begin{array}{ll} 0 & t_{i}\leq t\\ 1 & t_{i}>t \end{array}$$ (9)

The statistical values of all sub-blocks form a sequence of binary eigenvectors. The statistical order is from the inside out, from 0° to 360°. That is, for each sector that is divided, statistical block binary features extraction is performed from the inside out. This statistical method makes it possible to complete the rotation matching of the target area only by bit-shifting of the bit string in the subsequent feature matching process; thereby it can ensure the rotation invariance. For a target area with *M* rings and each divided into *K* equal parts, the resulting feature sequence is:

$$SB(T(x,y))=\{b_{1},\cdots ,b_{M},\cdots ,b_{i},\cdots ,b_{K\times M}\}$$ (10)

**Fig 5:**
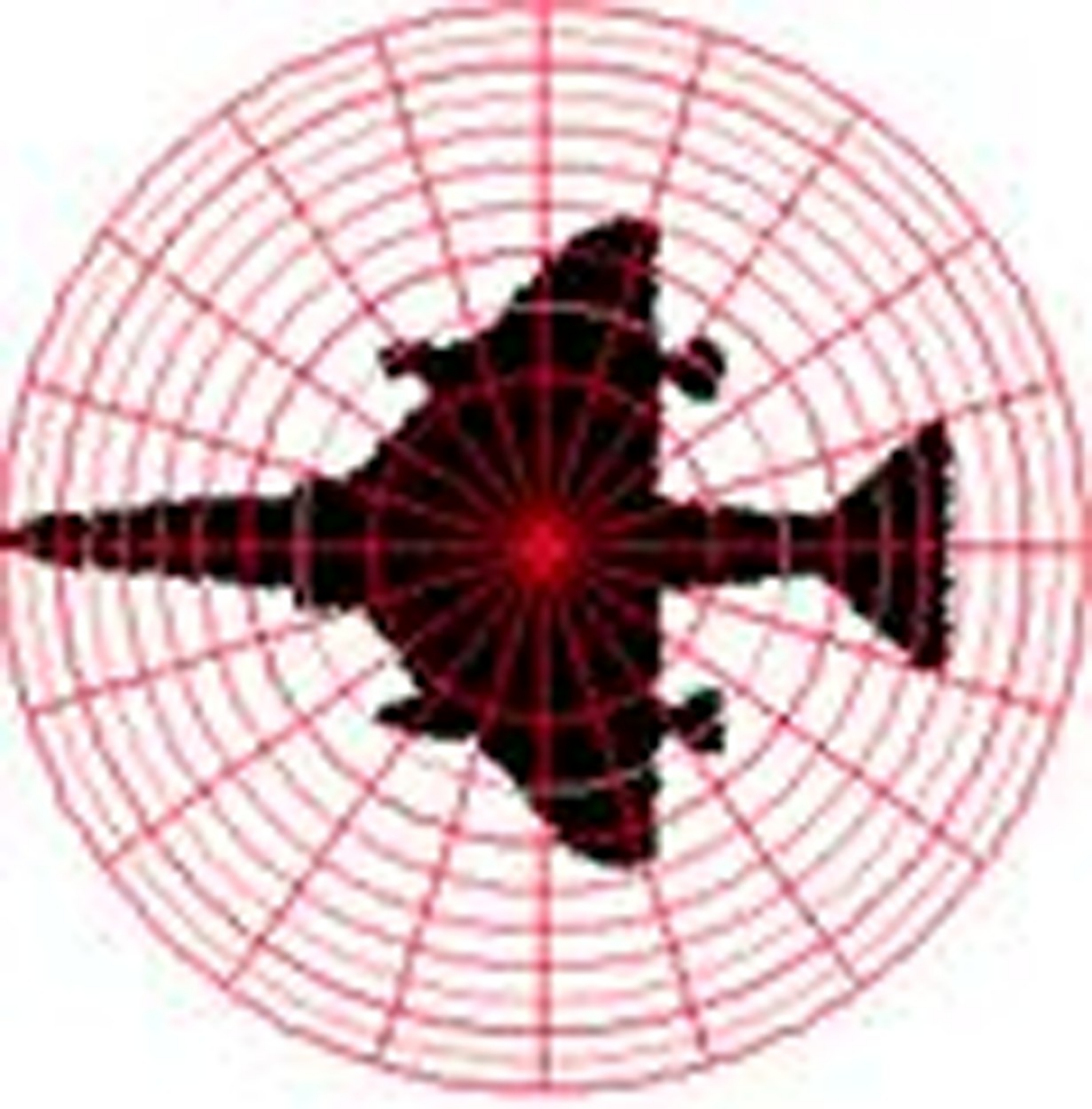
Block partition.

Taking into account both efficiency and performance, we set *M* = 10, *K* = 20, *t* = 0.1. Fig 5 shows an example of dividing the concentric rings into blocks. The area of each divided block is the same.

#### 3.2.2 Description of border features

**Fig 6:**
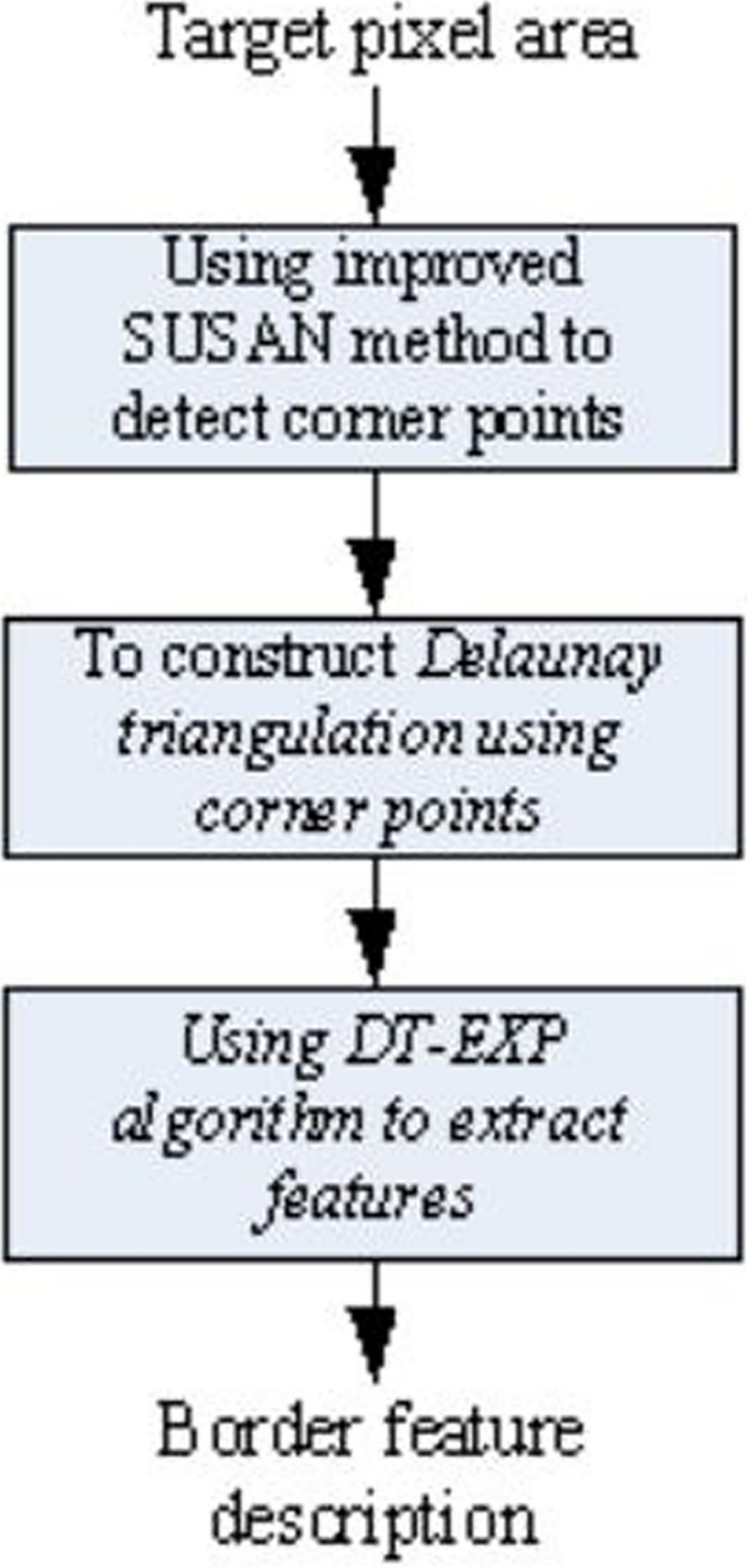
Border feature extraction.

The process of border features extraction is shown in Fig 6. On the basis of obtaining the target pixel area, we first use the improved SUSAN method to detect the corner points, and eliminate the error corner. Then the Delaunay triangulation, which has the powerful shape-body reconstruction capability, is used to perform the Delaunay triangulation on the detected corner points. Finally, we use the DT-EXP algorithm to extract features of Delaunay triangulation, and form a border feature description of the shape. Details of the border feature extraction process are as follows.

(1) The first is the corner detection using the improved SUSAN algorithm. The Corner Point is an important local feature of the image. To a certain extent, it can determine the shape of the target in the image. At present, there is no uniform definition for corner points. It is generally believed that corner points is the intersections of two or more relatively straight-line. There are many kinds of corner detection methods. Here are some examples. We can determine the corner points by calculating the curvature using the contour points getting from chain code tracking. What’s more, the corner points can also be detected by using the directional derivatives. using image gray information directly is another example[20]. The use of corner points for the extraction of the underlying features not only reduces the redundant information in the image features, but also simplifies the feature extraction process and obtains rich image local features.

**Fig 7:**
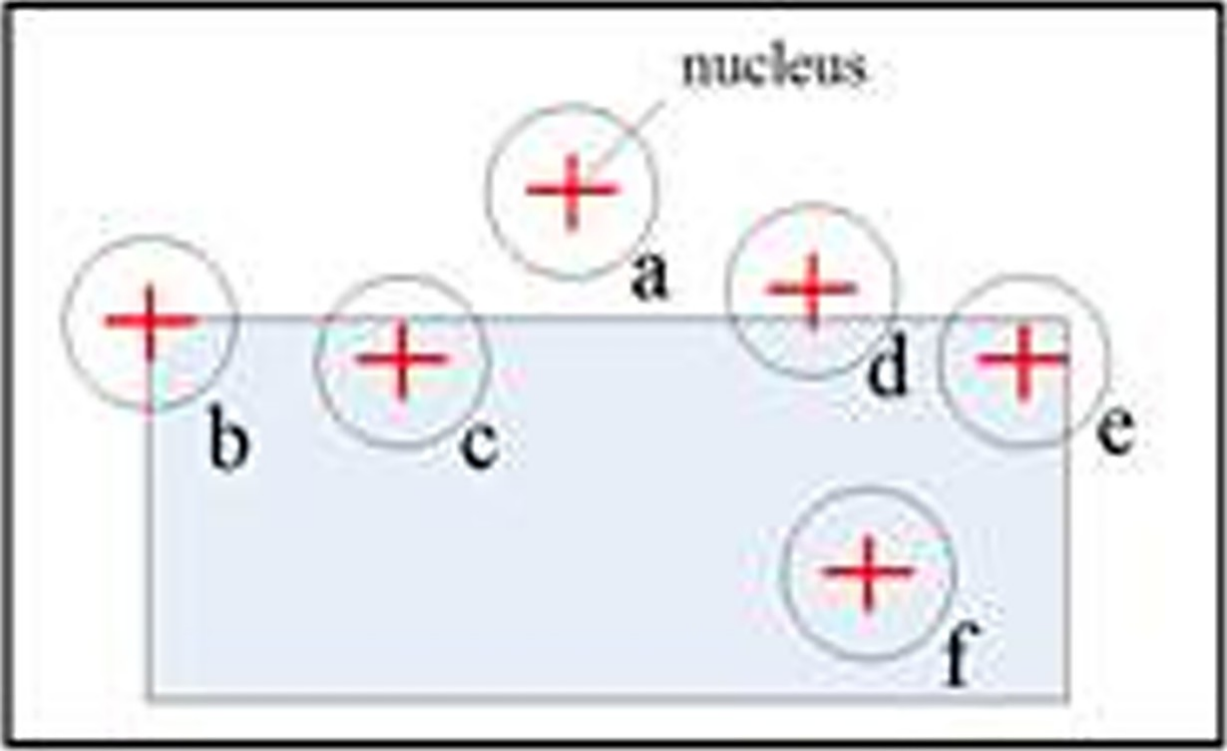
SUSAN.

Smith and Brady[21] introduced a simple and intuitive method, SUSAN, which is insensitive to noise and the accuracy and speed of the algorithm are both acceptable. The principle of the SUSAN principle is shown in Fig 7. A circular template is used to traverse the image. If the difference between the gray value of any other pixel in the template and that of the central pixel (nucleus) is less than a certain threshold, the point is considered to have the same (or close) gray value as the nucleus, and a region composed by pixels satisfying such a condition is called a Univalue Segment Assimilating Nucleus (USAN). The formula is as follows:

$$c(r,r_{0})=\{\begin{array}{cc} 1 & if\left|I(r)-I(r_{0})\right|\leq t\\ 0 & f\left|I(r)-I(r_{0})\right|>t \end{array}$$ (11)

c(*r*,*r*_0_) is the discriminant function used to determine whether a pixel within the template belongs to the USAN area; *I*(*r*_0_) is the grayscale value of the central pixel (nucleus); *I*(*r*) is the grayscale value of any other pixel within the template; and *t* is the grayscale difference threshold, which affects the number of corner points detected. As *t* decreases, we can obtain more fine-grained changes in the image, and thus a relatively large number of detections. The threshold *t* must be determined based on factors such as image contrast and noise. The size of the USAN area can be expressed by the following equation:

$$n(r_{0})=\sum_{y\Leftrightarrow y_{0}}c(r,r_{0})$$ (12)

When the template is completely in the background or the object, the USAN region is the largest (as a and f in Fig 7); when the template is moved to the target edge, the USAN region gradually becomes smaller (as c, d, e in Fig 7); when the center of the template is in the corner position, the USAN area is very small (b in Fig 7). After obtaining the size of the USAN region corresponding to each pixel, we can generate the initial corner response using the following equation:

$$R(r_{0})=\{\begin{array}{cc} g-n(r_{0}) & if\;n(r_{0})<g\\ 0 & else \end{array}$$ (13)

*g* is the geometric threshold, which affects the shape of the detected corner. The smaller is *g*, the sharper the detected corner. The last stage of the SUSAN criterion is to find the local maximum of the initial corner response that is, obtaining the final corner position using the non-maximal suppression processing. Non-maximal suppression, as the name implies, is a process of retaining the value if the initial response of the center pixel is the maximum value in the local area, and deleting it otherwise, so that the maximum value of the local area can be obtained.

We propose an improved SUSAN corner detection algorithm. The improvement is mainly reflected in three aspects: Firstly, the space for searching candidate corner points is limited, which makes the algorithm more efficient; secondly, the adaptive local threshold is used instead of the global threshold, which improves the performance of the algorithm. Finally, according to the degree of contribution of the corner points, the wrong and fuzzy corners are excluded.

Based on the fact that the corner points are located at the ends of the edges, it is not necessary to find the corner points on the entire image; we just need to find it on the edges. Therefore, the algorithm of this paper first uses the Sobel operator to detect edges in the preprocessing stage. Compared with the traditional SUSAN algorithm, this method can more effectively filter candidate corner points.

The choice of gray-diff *t* threshold affects the number of corners detected, and different *t* values should be chosen for images with different contrast and noise conditions. However, even for the same image, it is not appropriate to use the same threshold for the entire image because the contrast and noise conditions are not the same for each area of the image. For this paper, we obtain the adaptive local threshold for each template region using the largest inter-class variance Otsu algorithm. Otsu algorithm divides the pixels of the image into two types *C*_0_ and *C*_1_ according to the thresholds, and then calculates the intra- and inter-class variances of *C*_0_ and *C*_1_ respectively. The selected threshold makes inter-class variances maximized. The Otsu algorithm is based on the fact that the variance among pixels belonging to the same object in the image should be small, and the average difference between different regions should be maximized.

After the non-maximal suppression processing, we get the final corner position after finding the local maximum of the initial corner response. However there are still wrong corner points among the corners we get, such as circular or inconspicuous ones. The following criteria are used to eliminate them. In general, a well-defined corner point should have a sharp corner and two relatively long sides. We can easily distinguish the authenticity of a corner if we know the angle of each vertex and the two relative side lengths. We remove the wrong corners based on the following corner-point significance measure:

$$R_{i}=\frac{\theta_{i}\times L_{i}\times L_{i+1}}{L_{i}+L_{i+1}}$$ (14)

**Fig 8:**
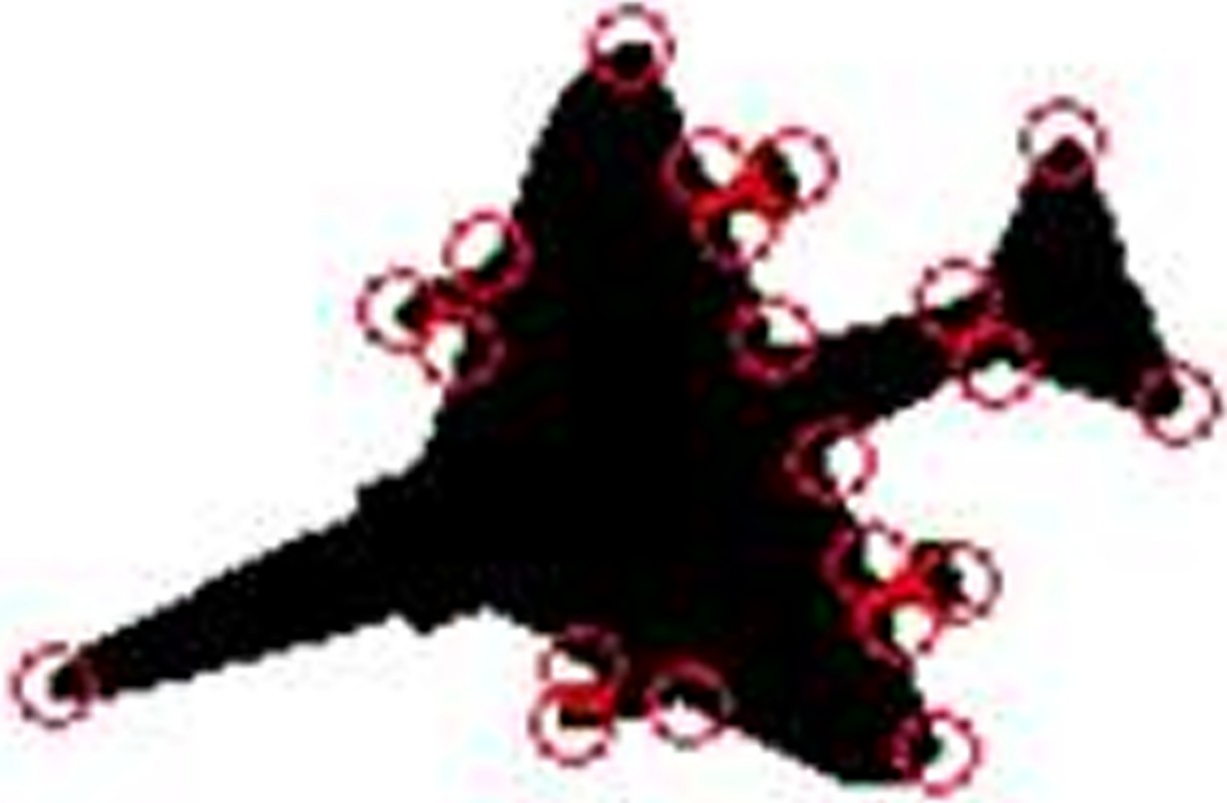
Corner detection.

*θ*_*i*_ represents for the angle at the point *P*_*i*_, and *L*_*i*_ and *L*_*i*+1_ are two opposite side lengths, which have been standardized according to the total length of the entire polygon curve. The larger the value of *R*_*i*_, the greater the contribution of the corner point to the shape of the curve. If both the angle *θ*_*i*_ and the two opposite sides are small, then the point will be excluded as a wrong corner when the value is below a certain threshold. Fig 8 shows an example of corner detection using the modified SUSAN algorithm.

(2) Secondly, we use corner points to construct the Delaunay triangulation. We first define the Voronoi diagram, and then we define the Delaunay diagram by the Voronoi diagram. Finally, we give the construction steps of the Delaunay diagram.

**Definition 1** Voronoi graph[22]: Given a set of points *V* = {*v*_1_,*v*_2_,⋯,*v*_*n*_}, the Voronoi region *V*_*i*_ of the point *v*_*i*_ is the set of all points that are closer to the point *v*_*i*_ than to other points in the set of points. The formula is as follows:

$$V_{i}=V(v_{i}:V)=\left\{v\in R^{d}|d(v,v_{i})<\underset{i\neq j}{\min}d(v,v_{j})\right\}$$ (15)

**Fig 9:**
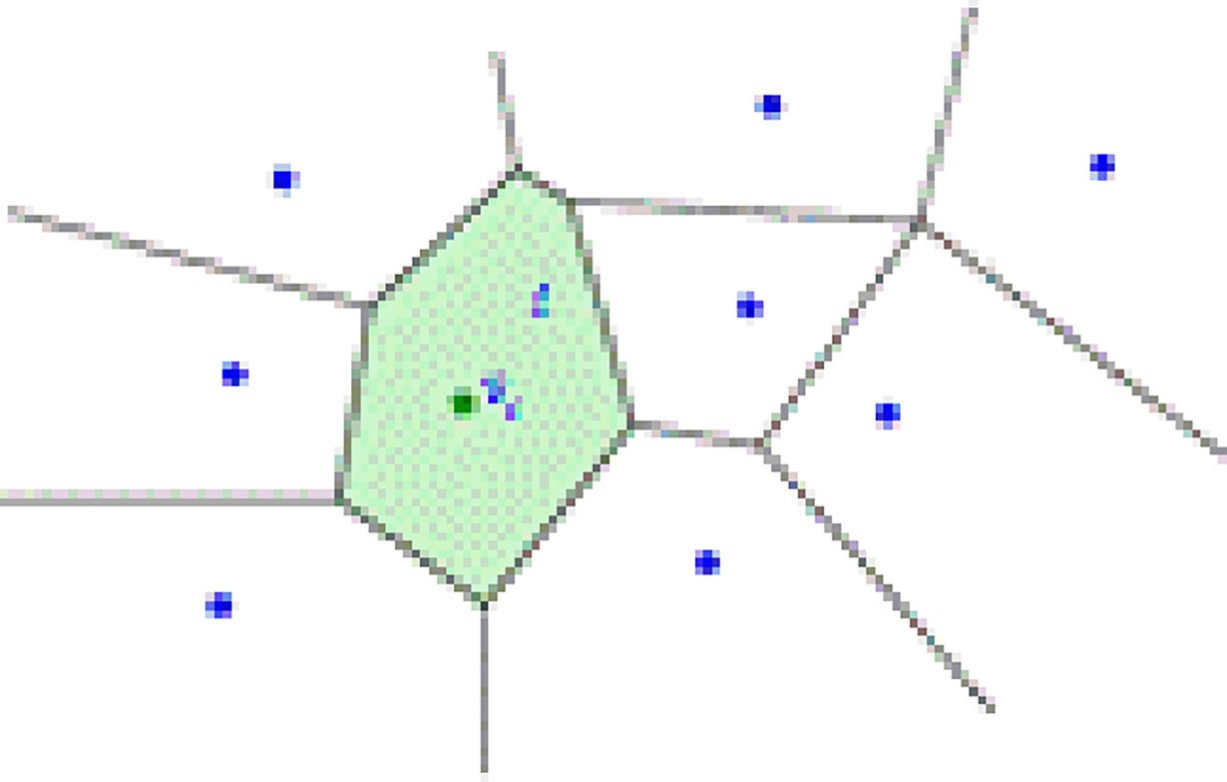
Voronoi.

In general, *d* is the Euclidean distance, and *V* = {*v*_1_,*v*_2_,⋯,*v*_*n*_} is called the Voronoi diagram, as shown in Fig 9.

The Voronoi diagram divides the plane into several regions, where the points are closer to *v*_*i*_ than those of other points in *V*. Given two points *v*_*i*_ and *v*_*j*_, the set of points that are closer to *v*_*j*_ than *v*_*i*_ is exactly the half-plane containing *v*_*j*_ that is contained by the two-point vertical bisector.

**Definition 2** Delaunay graph: It can be defined by the Voronoi diagram. The vertex set of the Delaunay graph is the point set *V*. And there is an edge of *v*_*i*_ and *v*_*j*_ if and only if the Voronoi cell $V_{i}$ containing *v*_*i*_ and the Voronoi cell $V_{j}$ containing *v*_*j*_ have shared edges.

**Fig 10:**
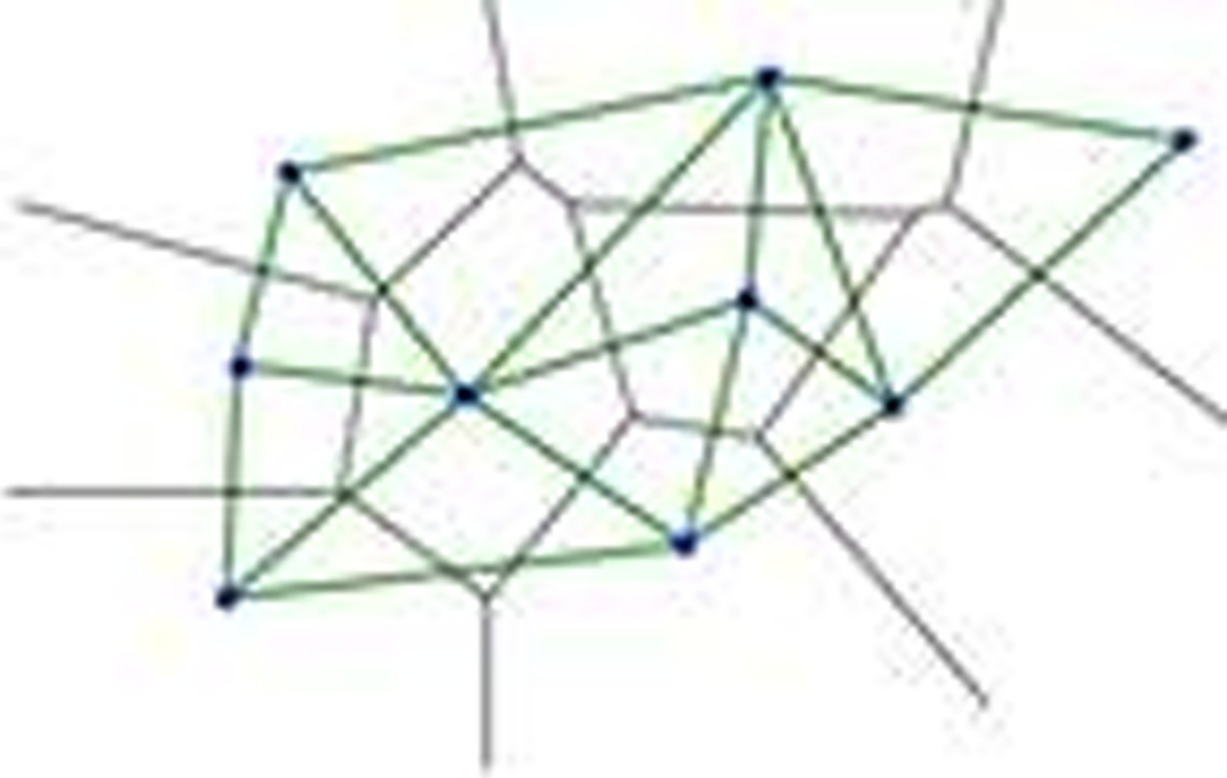
Voronoi linked arc.

**Fig 11:**
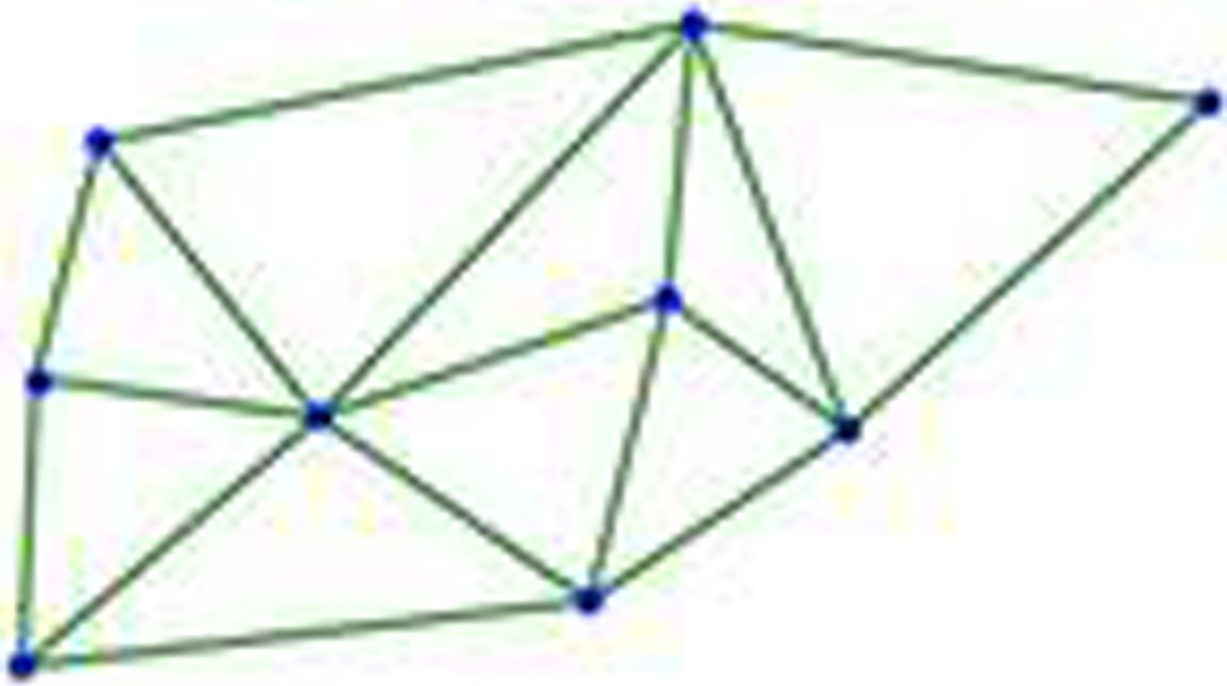
Delaunay.

As shown in Fig 10, an arc is added between each pair of points (they share an edge in the Voronoi diagram), and then all the arcs are drawn into a straight line to obtain the plot of the embedded plane. The resulting graph is a Delaunay plot over the original n points, as shown in Fig 11. According to the Delaunay theorem [23], such a linear dual graph is a triangulation of *V*. The Delaunay diagram and the Voronoi diagram are both dual.

The outer boundary of the Delaunay graph is a convex polygon, which has two important properties[24]: 1.null Circumcircle property. In the Delaunay graph formed by the set of points *V*, the circumscribed circle of each triangle does not contain any other arbitrary point in *V*. That is, the Delaunay graph is unique, and there is no situation of having more than four points in a single circle. 2. The largest minimum angle property. In the triangulation network that is formed by the point set *V*, the minimum angle of the triangle in the Delaunay graph is the largest of all possible triangulations.

**Fig 12:**
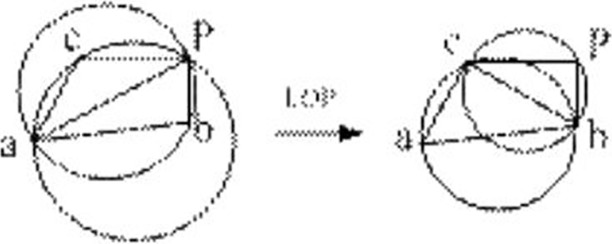
LOP algorithm.

There are three main methods of triangulation of the delaunay graph: triangulation growth method, point-by-point interpolation method, and segmentation and merging method. We introduce only the point-by-point interpolation method. The steps for constructing a Delaunay graph are as follows:

1. Define an initial polygon containing all data points;
2. Create an initial triangulation in the initial polygon and then iterate the following steps until all data points are processed;
3. Insert a data point *P*, find the triangle containing the point *P* in the triangulation, connect the point with the three vertices of the triangle, and generate new triangles;
4. Use the Local Optimization Procedure (LOP) algorithm to optimize the triangulation. The basic idea of the algorithm is to perform analysis and processing on the quadrilateral consisting of two common edges. If the circumscribed circle of one of the triangles contains the fourth vertex, then we exchange the diagonal of the quadrilateral. The optimization process of the LOP algorithm is shown in Fig 12.

**Fig 13:**
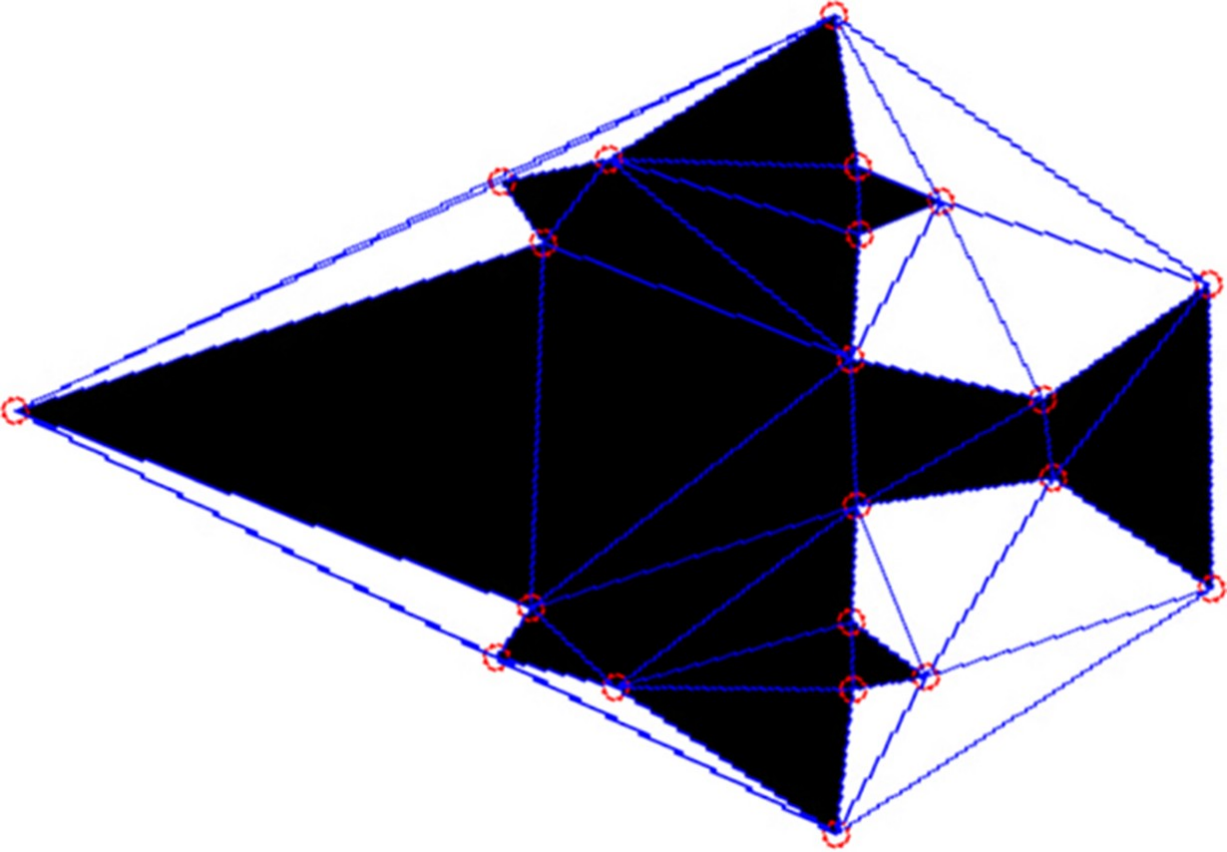
Delaunay triangle mesh.

For a set with *n* points, the time complexity of constructing the Delaunay triangulation algorithm is *O*(*n*log*n*)^[39]^. Fig 13 is an example of constructing a Delaunay triangulation based on the corners detected in Fig 8.

(3) The Final step is the feature extraction of Delaunay triangulation. In the Delaunay triangulation, the maximum angular range of each triangle is [60°, 180°) and the minimum is (0°, 60°). Suppose that the maximum and minimum interior angles of the triangle are *α* and *β* respectively, and then for a small quantization interval *η*, the triangle can be represented by a quantified value:

$$T_{i}=f(\frac{\alpha_{i}-60}{\eta })\times \frac{60}{\eta }+f(\frac{\beta_{i}}{\eta })$$ (16)

**Fig 14:**
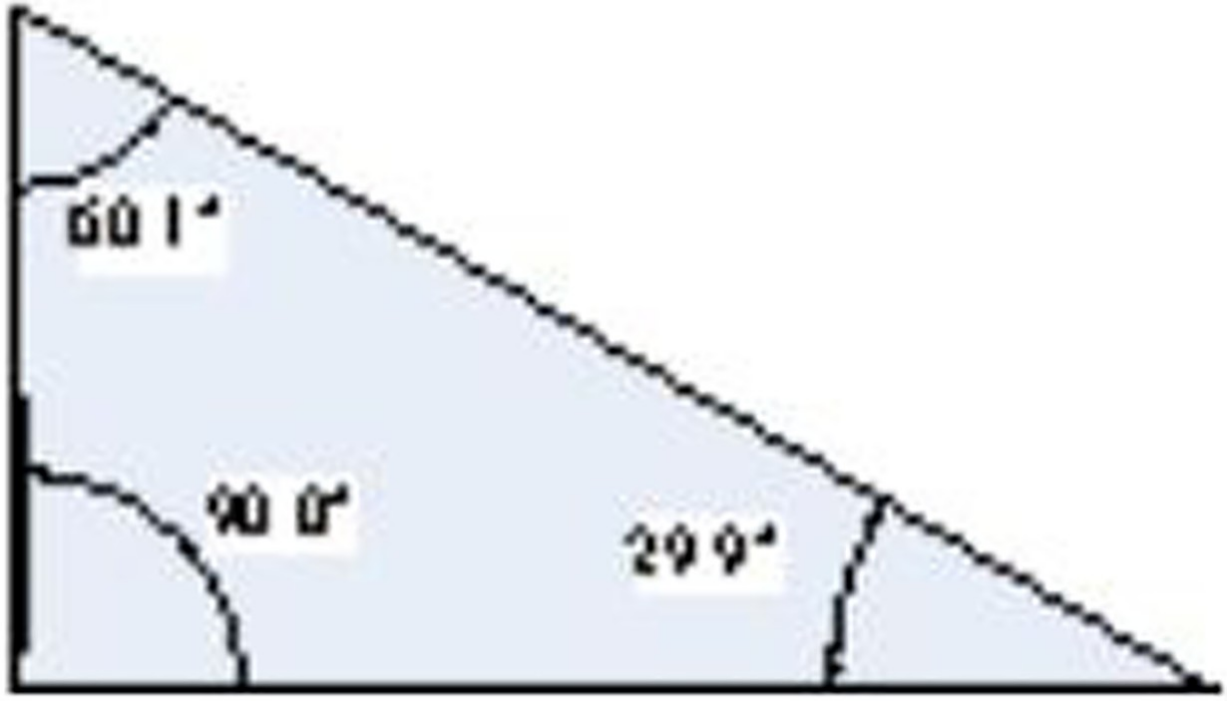
Triangle quantitative.

*f*(*x*) is used to transform the element value to the nearest integer; αi−60η and βiη are the quantized values of the maximum and minimum interior angle respectively; 60η is the number of quantization intervals of the minimum interior angle. Suppose *η* = 10°, then the quantified value of the triangle in Fig 14 is: $T=f(\frac{90.0-60}{10})\times \frac{60}{10}+f(\frac{29.9}{10})=21$

For each triangle in the Delaunay triangulation, we obtain both the quantized value and the largest edge corresponding to the largest interior angle to uniquely represent a triangle. The following DT-EXP algorithm is a process of feature extraction of Delaunay triangulation.

Algorithm: DT-EXP

Function: Feature extraction for Delaunay triangulation

Input: Delaunay Triangulation

Output: A series of quantized values in descending order and corresponding maximum edges

1. For each triangle in the Delaunay triangulation, quantify the triangle using Eq (7);

2. For each triangle in the Delaunay triangulation, obtain its length of the maximum edge;

3. Sort the sequence of quantized values in descending order, and adjust the sorting of side lengths according to the sorting result;

4. Output a series of quantized values in descending order and corresponding maximum edges.

### 3.3. Shape feature matching

For a trademark image *I*, its final shape features can be described as:

$$F_{I}=\left\{B_{I},A_{I},E_{I}\right\}$$ (17)

*B*_*I*_ represents the binary feature sequence obtained by performing the region description method on image *I*,

$$B_{I}=\{b_{1}^{I},b_{2}^{I}\cdots ,b_{m}^{I}\}$$ (18)

$b_{j}^{I}$ represents the final statistical value of each block area with *j* = 1,2,⋯*m*, which is in the range of 0 or 1. *m* is the number of block areas in the target area. According to the method of dividing parameters in this paper, *m* is determined with its number to be 200. *A* and *E* represent the quantized value sequence and the maximum edge sequence obtained by the border feature description method, respectively.

$$\{\begin{array}{l} A_{I}=\left\{a_{1}^{I},a_{2}^{I},\cdots ,a_{n}^{I}\right\}\\ E_{I}=\left\{e_{1}^{I},e_{2}^{I},\cdots ,e_{n}^{I}\right\} \end{array}$$ (19)

We use $\left\{a_{i}^{I},e_{i}^{I}\right\}$ to represent triangles in a Delaunay triangulation, where $a_{i}^{I}$ represents the quantized value of the triangle, and $e_{i}^{I}$ represents the length of the maximum side of the triangle, *i* = 1,2,⋯*n*, and *n* is the number of triangles in the triangulation. Its value is non-deterministic.

Based on the features extracted above, the feature matching process is divided into three steps, regional feature matching, border feature matching and fusion of feature matching results.

(1) The first is the regional features matching. For the two trademark images *F*_*I*_ and *F*_*J*_, suppose that the regional features are *B*_*I*_ and *B*_*J*_ respectively and the feature lengths are all *m* = *K*×*M*. The region feature matching algorithm is as follows.

Algorithm: R-MATCH

Function: similarity measures for the regional features of the two trademark images

Input: Regional features of the two trademark images *B*_*I*_, *B*_*J*_

Output: similarity score *S*_*R*_

1. For *i* = 1,⋯,*K*−1, perform 2~4 steps;

2. Perform M-bit displacement operations on b *i* times, and then form new features;

3. Calculate the number of non-zero similarities between new features *Rotate*(*B*_*I*_) and *B*_*J*_: *S*_*i*_ = ∪(*Rotate*(*B*_*I*_)∧*B*_*J*_)

4. Calculate the normalized score of the two trademark images: $S_{iR}=\frac{2\times S_{i}}{\cup (B_{I})+\cup (B_{J})}$

5. Compare and output the maximum of all rating values *S*_*iR*_: *S*_*R*_

In the algorithm, "∧" is the bitwise AND operator, and the "∪" operator is used to add the feature series in bits. Performing the displacement operation on the binary shape feature string is actually a rotation operation of the trademark object. Through such a cyclic movement comparison process, the trademark rotation invariant matching process can be effectively realized. The score range of the last output of the algorithm is [0, 1], which makes the similarity between different trademark images have better comparability.

(2) Then we perform the matching of the border features. For the two trademark images, we suppose that the border features are {*A*_*I*_,*E*_*I*_} and {*A*_*J*_,*E*_*J*_} respectively, and the feature lengths are *n*_1_ and *n*_*2*_ respectively. Firstly, a similar triangle pair is obtained by comparing the quantized values; Secondly, we further determine whether the similar triangle pair is the same according to the largest edge, and if so, we increase the score value by one. The specific algorithm process is described as follows.

Algorithm: B-MATCH

Function: Similarity measures for the border features of two trademark images

Input: Border features of the two trademark images {*A*_*I*_,*E*_*I*_}, {*A*_*J*_,*E*_*J*_}, Similarity threshold *T*

Output: similarity score *S*_*B*_

1. Set initial value, *i* = 1, *j* = 1,*S* = 0;

2. Repeat the following steps 3, 4, 5 and 6 when *i*≤*n*_1_ and *j*≤*n*_2_.

3. If $a_{i}^{I}==a_{j}^{J}$, which means the quantized values of the triangles are equal, then we think it’s a similar triangle pair in a preliminary estimate;

4. If the two triangles are interpreted as similar triangles, and the maximum edges are $e_{i}^{I}$ and $e_{j}^{J}$ respectively, then *S* = *S*+1,*i*++, *j*++ when $\left|e_{i}^{I}-e_{j}^{J}\right|<T$.

5. If $a_{i}^{I}>a_{j}^{J}$, then *i*++, that is, the quantized value sequence moves one step forward and selects the quantized value of the small point;

6. If $a_{i}^{I}<a_{j}^{J}$, then *j*++;

7. Standardize the score *S*: $S_{B}=\frac{2\times S}{n_{1}+n_{2}}$;

8. Output similarity rating value *S*_*B*_.

The similarity threshold *T* in the algorithm needs to be selected based on the size of the trademark image, and generally we set the image width to be 0.1. The score range of the last output *S*_*B*_ of the algorithm is [0, 1], which makes the similarity between different trademark images have better comparability.

(3) Finally, we synthesize the results of the matching of border features and regional features. The following formula shows the fusion process using the weighting method:

$$Sim=w_{1}S_{B}+w_{2}S_{R}$$ (20)

There are some problems with this method: On the one hand, the weighting parameters are not easy to determine; on the other hand, in the shape description, shapes with similar regional features may have quite different border features. This is because the boundary-based method is not suitable for describing complex shape. It is sensitive to local changes. Therefore, there may be a wide gap in data, so it’s not appropriate to use the same weight for all matching results.

We use the following criteria to synthesize feature matching results. If one of border features and regional features is similar, then the two trademark patterns are considered the same. Since the values of *S*_*B*_ and *S*_*R*_ are already normalized in the interval range [0, 1], we can define the final similarity score as follows:

$$Sim=\max(S_{B},S_{R})$$ (21)

Its range of values is also [0, 1]. We named the method BR-MATCH, which combines two feature matching methods: B-MATCH and R-MATCH. Finally, the system sorts and output the database image list according to similarity scores.

## Experimental results and discussion

The following experimental databases used in the present study were employed from the research image dataset provided by Xiamen Tuxingtianxia Info Technology CO.,Ltd. and the company guarantees that these trademark images can be legally used to extensively evaluate our BR-MATCH Method in this study.

In the first experiment, three different methods and two common methods are used in a simple shape database to facilitate a clear comparison of the performance of different algorithms. In the second experiment, we construct a new data set by rotating, scaling and translating the conventional trademark image to test the anti-interference ability of the algorithm. In the third experiment, we make it more difficult to deal with the data set, and perform various distortions on the data set, which further verifies the robustness of the algorithm. Finally, in the fourth experiment, the proposed algorithm and the conventional algorithm are applied to the real large data set of trademark, and the feasibility and effectiveness of the algorithm in practical application are verified.

### 4.1. Shape image retrieval performance evaluation

**Fig 15:**
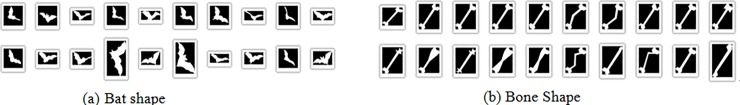
Examples of the CE-SHAPE-1 database.

First, the shape-image retrieval experiment was performed using the MPEG-7 core experimental database CE-SHAPE-1 to evaluate the performance of the BR-MATCH algorithm. The database CE-SHAPE-1 contains 70 different types of shapes, each containing 20 similar shape bodies. These shape bodies are usually a distorted basic shape, and the entire data set has a total of 1400 images. For example, as shown in Fig 15, (a), (b) respectively show the bat and bone classes of the data set, each containing 20 similar images.

Each image is retrieved as a query image (a total of 1400 retrievals are performed). The retrieval rate is evaluated by the Bull's Eye Percentage (BEP) [4] performance index. It represents the proportion of images in the same category in the first 40 images retrieved. Assume the actual number of matches for each query result, is *n*_*i*_, and then the total actual number of matches will be $\sum_{i=1}^{1400}n_{i}$. The number of ideal correct matches for each query result is 20, so the total number of ideal correct matches is 28000(1400×20 = 28000). The overall retrieval rate is defined as the ratio of the number of actual correct matches to the number of ideal correct matches. The formula is as follows:

$$BEP=\frac{\sum_{i=1}^{1400}n_{i}}{28000}$$ (22)

Below is the performance comparison of the following method: regional-based feature description and matching method R-MATCH, boundary-based feature description and matching method B-MATCH, the matching boundary and regional features fusion method BR-MATCH, and two other well-known methods (Hu moments and Zernike moments methods[25]). Table 1 shows the corresponding retrieval rates for the above methods. As can be seen from the table, the retrieval performance of the three methods in this paper is superior to the Zernike moment and Hu moment method. The BR-MATCH method combines the advantages of the B-MATCH method and the R-MATCH method, and chooses the best among the local optima. Therefore, the final performance is greatly improved, which is superior to the other two methods.

**Table 1:** BEP retrieval performance evaluation.

	*Hu*	*Zernike*	*B-MATCH*	*R-MATCH*	*BR-MATCH*
**BEP performance**	0.351	0.458	0.516	0.551	**0.681**

### 4.2. Trademark affine transformation experiments

**Fig 16:**
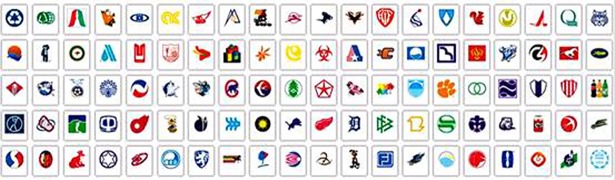
Transform invariant search experiment for 100 random selected images.

**Fig 17:**
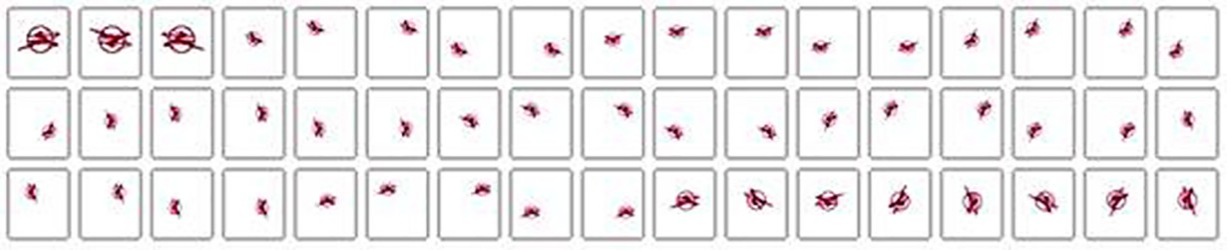
Translate, scale, and rotate the trademark.

One hundred images of different shapes were randomly selected from the image database and the BR-MATCH method was used to carry out invariant retrieval experiments. The selected trademark image is shown in Fig 16. Each image is processed with any combination of the following transformations to obtain 50 similar images of the original image respectively:

1. Translation, translate the image target object to any position in the image;
2. Scaling, magnify or reduce the image at any magnification
3. Rotate, rotate the image at any angle;

This results in a set of images containing 100 classes and 50 images per class. Fig 17 shows the result of transforming one of the images. Among them, the first image is the source map, and the other 50 are the transformation results.

The 5000 images are added to the original image library to form a new image library. Each search retrieves an image from each class as a sample. The search results are sorted and output according to the degree of similarity, and the first 100 result images are directly displayed. The similarity between the sample image and itself is 1, and the first image returned is the sample itself. Using the BEP performance index to evaluate the retrieval rate of the BR-MATCH method, the average retrieval rate reaches 0.96. This shows that the BR-MATCH method has good invariance to translation, scaling and rotation.

**Fig 18:**
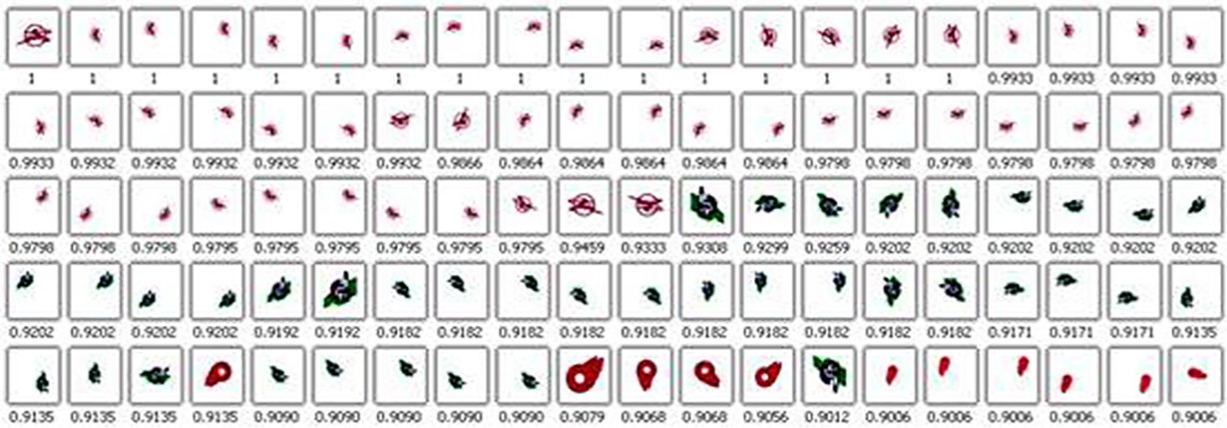
Transformed image search by BR-MATCH.

Fig 18 shows the result of invariance experiments using the BR-MATCH method for the same sample image above. It can be seen that images of the same kind as the sample images are retrieved and arranged at the front of the search result. The degree of similarity is given below the image. The value interval is [0, 1], and the bigger the value is, the more similar the image is to the sample image.

### 4.3. Trademark geometry deformation experiment

**Fig 19:**
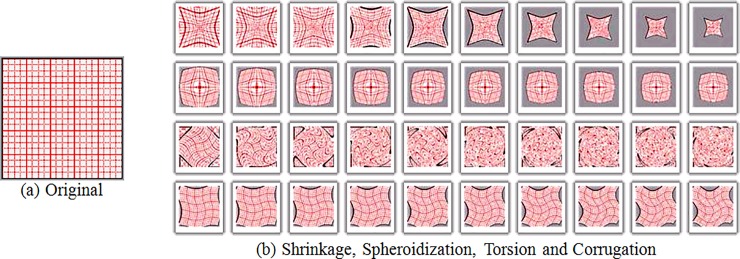
Geometry deformation diagram.

**Fig 20:**
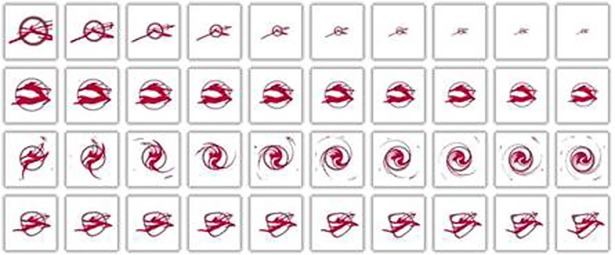
Shrinkage, spheroidization, torsion and corrugation for trademark.

In order to examine the ability of the BR-MATCH method to retrieve geometrically deformed images, we first perform the geometric deformation of the 100 images in Fig 16. Geometric transformations include: shrinkage, spheroidization, torsion, and corrugation. Fig 19 shows a schematic diagram of various geometric deformations, in which Fig 19 (A) is the original image, and the four lines in Fig 19 (B) are the results of different degrees of deformation including the contraction, spheroidization, twisting, and corrugation to the corresponding original image. Fig 20 shows the result of the geometric deformation of the above image. This results in a set of images containing 100 classes and 40 images per class.

The 4000 images are added to the original image library to form a new image library. Each search retrieves one image from each class as a sample. The retrieval results are sorted and output according to the degree of similarity, and the first 80 result images are directly displayed. The similarity between the sample image and itself is 1, and the first image returned is the sample itself. Using the BEP performance index to evaluate the retrieval rate of the BR-MATCH method, the average retrieval rate is 0.35. This shows that although the BR-MATCH method has a certain ability to retrieve geometric deformation images, it is very limited and the retrieval results are not satisfactory. Fortunately, the deformation of the trademark image is less likely to occur, and it is always slight even if it occurs.

**Fig 21:**
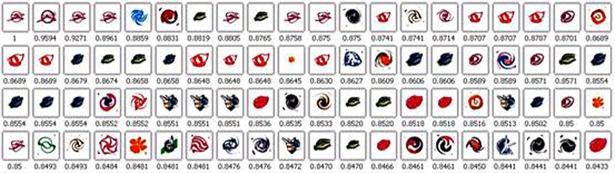
Transformed image search by BR-MATCH.

Fig 21 shows the results of the deformation retrieval using the BR-MATCH method for the same sample image above. It can be seen that the slightly distorted images of the same type as the sample image can be retrieved and arranged at the front end of the search results, and Images with large distortion are sometimes sorted later in the resulting image. The degree of similarity is given below the image. The value interval is [0, 1], and the bigger the value is, the more similar the image is to the sample image.

### 4.4. Performance evaluation of the classification trademark database

In order to further evaluate the performance of the full-image trademark image retrieval method proposed in this article, an experiment was conducted with the classification trademark database containing 2000 images. Trademark images are collected from the Internet and have been divided into 20 categories. The system uses the precision ratio and recall ratio as performance indicators to evaluate the retrieval performance.

**Fig 22:**
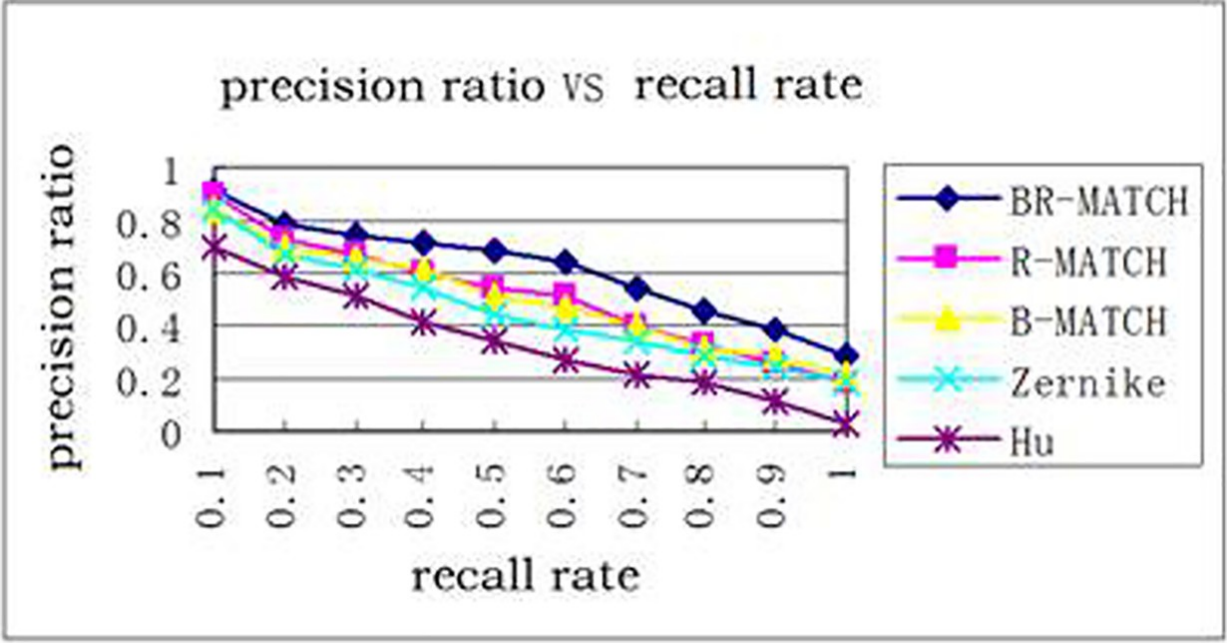
Evaluation of retrieval performance.

**Fig 23:**
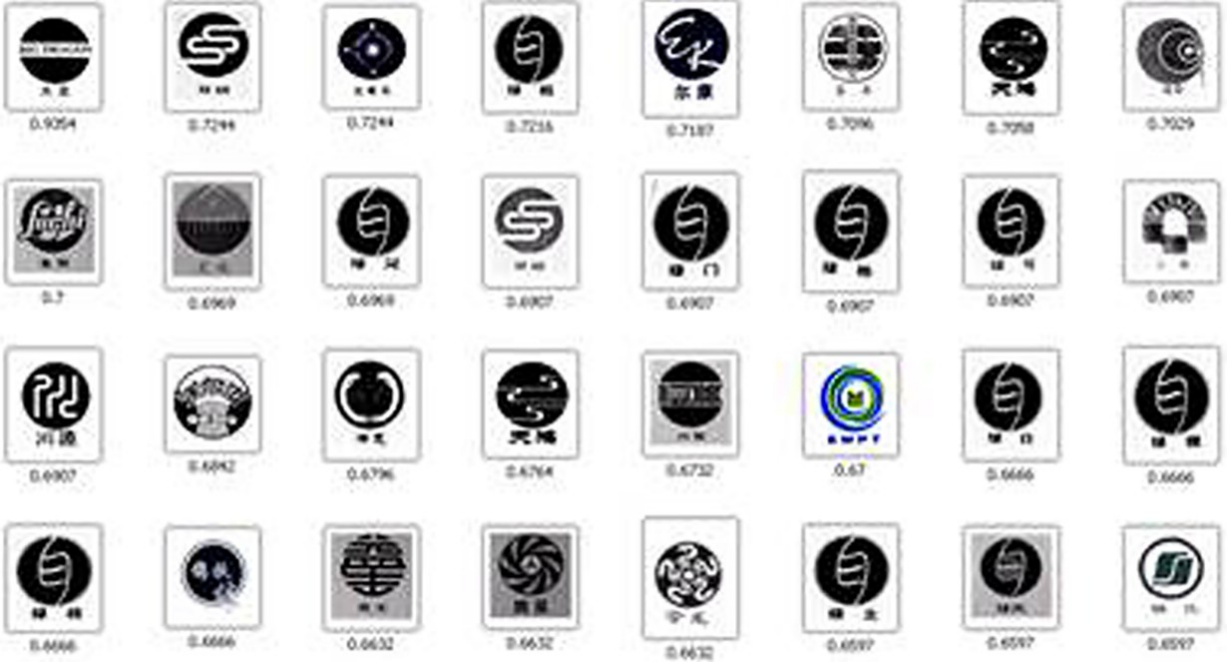
A retrieval demonstration via BR-MATCH.

For performance evaluation, 100 trademark images were randomly selected from the database and these images were used as query samples. We still use the five methods in Section 3.1 as the comparison method. Fig 22 shows the average precision ratio curve under different recall rates. It can be observed that the boundary and region feature fusion method BR-MATCH has satisfactory retrieval performance and its performance is superior to the other four methods. Fig 23 shows an example of the result using BR-MATCH. The trademark image in the upper left corner is the source map. The retrieval results are arranged according to the degree of similarity, which is marked below the image.

## Conclusions

This paper proposes a full-image trademark image retrieval method based on regional and border feature fusion. Based on the target image extraction, this method describes the target regional and border features. The regional feature description is mainly based on the concept of partition block statistics. The region is divided into equal-area unit using concentric circles, and feature extraction is performed in each small block units. For the border feature description, we first detect corners, and then construct a Delaunay graph and extract features by combining the corner detected and the Delaunay triangulation reconstruction method. In the search process, the method also incorporates information such as trademark classification and trademark keywords.

The region-based method requires a lot of calculations, but it can be used to describe complex shapes and is insensitive to local variations. The boundary-based method is not suitable for describing complex shapes and is sensitive to local variations, but it has a lower computational cost. Since the region-based approach and the boundary- based approach are complementary, this paper synthesizes border-feature and region-feature description methods for content-based trademark retrieval.

The image retrieval experiment was carried out on CE-SHAPE-1 database containing 1400 MPEG-7 core experimental shape, a classification trademark database containing 2000 images, and a national trademark database containing approximately 4.89 million images. The experimental results show that the proposed approach combines the advantages of region and border feature description, and can choose the best among various local optimizations, which makes the retrieval result more effective, more in line with human visual perception, and improves the retrieval accuracy.
